# Coaxial electrospinning of liquid crystal-containing poly(vinylpyrrolidone) microfibres

**DOI:** 10.3762/bjoc.5.58

**Published:** 2009-10-23

**Authors:** Eva Enz, Ute Baumeister, Jan Lagerwall

**Affiliations:** 1 Institute of Chemistry – Physical Chemistry, Martin-Luther-University Halle-Wittenberg, Von-Danckelmann-Platz 4, 06120 Halle, Germany

**Keywords:** coaxial electrospinning, composite material, core-sheath fibres, liquid crystal, smectic phase

## Abstract

With the relatively new technique of coaxial electrospinning, composite fibres of poly(vinylpyrrolidone) with the liquid crystal 4-cyano-4′-octylbiphenyl in its smectic phase as core material could be produced. The encapsulation leads to remarkable confinement effects on the liquid crystal, inducing changes in its phase sequence. We conducted a series of experiments to determine the effect of varying the relative flow rates of inner and outer fluid as well as of the applied voltage during electrospinning on these composite fibres. From X-ray diffraction patterns of oriented fibres we could also establish the orientation of the liquid crystal molecules to be parallel to the fibre axis, a result unexpected when considering the viscosity anisotropy of the liquid crystal kept in its smectic phase during electrospinning.

## Introduction

**Figure 1:**
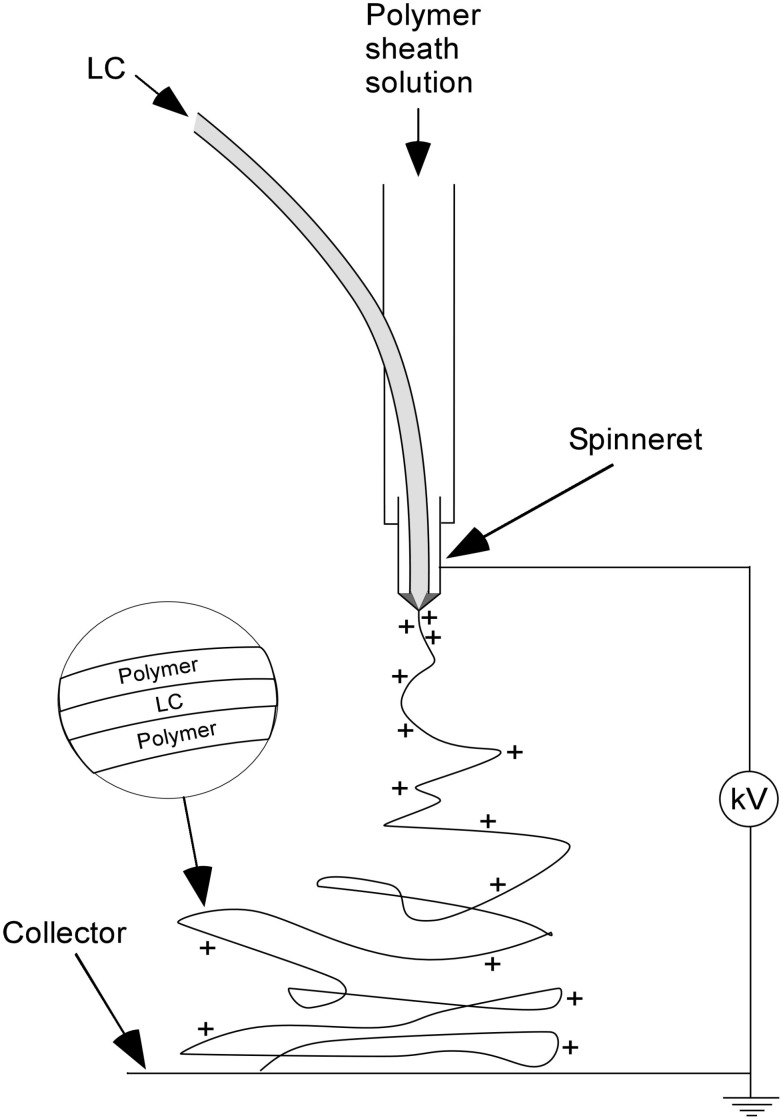
Schematic illustration of the main parts of the setup used in our lab for coaxial electrospinning and of the resulting composite fibres.

Electrospinning is a versatile process for producing nano- and microfibrous materials through electrostatic means. Even though the basic principles have been known for a long time and the first patents on electrospinning go back to 1902 [1–2], there has been a revival of interest since the beginning of the 1990s [3–6]. For laboratory scale purposes the simplest setup for electrospinning consists of three main components: a chargeable spinneret (e.g. a metallic needle) through which a polymer solution or melt is pumped, a grounded collector (e.g. an aluminium foil) and a high voltage power supply connecting spinneret and collector (see Figure 1).

The mechanism behind the electrospinning process is driven by an interplay of electrostatic forces and the surface tension of the polymer solution [5,7]. By applying a high DC voltage (several kV) between the spinneret and ground, surface charges are induced in a droplet of polymer solution protruding from the end of the spinneret, which then deforms into a so-called Taylor cone. When the electrostatic repulsion between the induced charges together with the coulomb force of the applied field become strong enough to overcome the surface tension, a liquid jet is ejected from the cone. This highly charged liquid jet experiences an electrostatic self-repulsion leading to a whipping motion and stretching process on its way to the collector, the latter leading to a dramatic reduction of fibre diameter. During this process most of the solvent must be evaporated, so that a stable, essentially dry fibre (the length of which in the ideal case is determined only by the time spinning is continued) is collected on a target substrate. This collection can be made on glass slides placed on the collector during electrospinning, resulting in a randomly oriented, nonwoven mat. Beside this, oriented fibres can also be achieved by modification of the target, e.g. by using two parallel electrodes as collector [8]. A recent review on different electrospinning setup designs is given in [9].

The morphology and final diameter of the resulting fibres can be influenced by several parameters, which can be divided into two categories: intrinsic properties of the polymer solution, and operational conditions. The most important are: type of polymer and its concentration; viscosity, electrical conductivity, polarity and surface tension of the solvent; applied electric field, distance between spinneret and collector, flow rate of the polymer solution and also the humidity and temperature of the surroundings, since they influence the evaporation of the solvent.

An interesting variation of electrospinning is the use of a spinneret comprising two coaxial capillaries, allowing two different liquids to be spun, one inside the other, leading to a composite fibre with core–sheath structure [10–12]. Recently it could be shown that also a room temperature nematic liquid crystal (LC) can be coaxially spun with a composite sheath of TiO_2_ and poly(vinylpyrrolidone) (PVP) [13]. Such composite fibres with a core exhibiting the responsiveness and special properties (in particular optical) that result from the unique combination of fluidity and long-range order of liquid crystals are interesting from different points of view. On the one hand, the LC can give the fibre new functionality, in particular sensitivity to temperature variations or to the application of electric and/or magnetic fields, on the other the strong confinement that can be achieved by the process can affect the LC phase sequence [14]. Electrospinning offers a cheap and simple way of studying such confinement effects systematically.

In this work we present our results on composites of PVP as sheath and the liquid crystal 4-cyano-4′-octylbiphenyl (8CB) as core produced by coaxial electrospinning. The latter LC exhibits a smectic phase (phase sequence: cryst. 20.5 SmA 32.0 N 39.2 iso.) in contrast to the LC used in our previous work, which formed only a nematic liquid crystal phase. After some general remarks on the properties of these composites, we show in the first part a systematic study on fibres with different content of LC core. Secondly we studied the influence of the magnitude of the applied voltage on the fibres. Finally we show X-ray investigations on these materials and discuss the orientation of the LC based on these results.

## Results and Discussion

**Figure 2:**
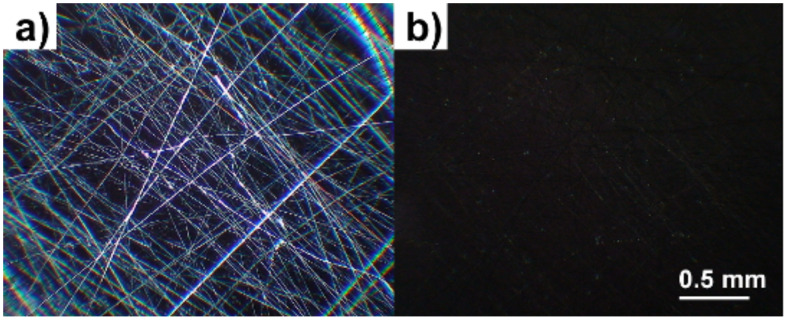
Polarizing optical microscopy photographs of 8CB-containing composite fibres; (a) SmA phase at room temperature; (b) isotropic state at 45 °C.

All fibres were spun at room temperature, i.e. with the LC in its smectic phase. In the resulting fibres the PVP sheaths were transparent and isotropic so that one could directly observe the birefringent liquid crystal core through a polarising optical microscope (POM) and follow the phase sequence (Figure 2). The fibres are thermally stable enough to allow repeated heating into the isotropic phase of the contained liquid crystal and cooling to room temperature again without any change in appearance. In differential scanning calorimetry (DSC) investigations only the transitions of the liquid crystal are found up to about 100 °C, where decomposition of the PVP sheath starts to take place. When fully dried the fibres also show good mechanical stability so that they can be pulled from the glass slide and folded or rolled together.

### Variation of the extent of LC filling

**Figure 3:**
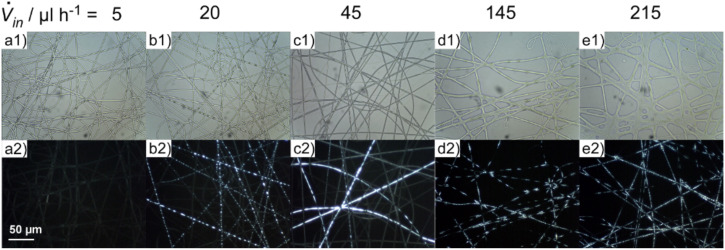
Microscopy photographs of characteristic samples when varying the flow rate of the liquid crystal 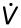
*_in_* while retaining the flow rate of the polymer solution constant. Upper row: sample seen without polarisers; lower row: same sample position seen between crossed polarisers.

In this part we will discuss characteristic examples of a series of composite fibres in which the flow rate of the polymer solution was kept constant at 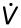
*_out_* = 125 µl h^−1^, while the flow rate of the LC was increased stepwise: 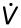
*_in_* = 5, 20, 45, 70, 115, 145, 190 and 215 µl h^−1^. In the upper row of Figure 3 non-polarising microscopy pictures of these samples are shown, while the lower row displays the corresponding POM pictures.

At the smallest flow rate presented here no liquid crystal is visible inside the resulting fibres (Figure 3a). At 20 µl h^−1^ droplets of LC appear in many of the fibres, the thicker ones being visible also in the non-POM photos as darker spots inside the fibres (Figure 3b). In the next sample (45 µl h^−1^, Figure 3c) the droplets have transformed into elongated segments. Such a transformation upon increasing flow rate of the inner fluid has been predicted by numerical modelling of the coaxial electrospinning process [15]. Depending on the exact spinning conditions either a bubbly slug or annular flow pattern may result. On the other hand there are still many parts of the fibre samples where no LC seems to be present at all.

When increasing the LC flow rate to 145 µl h^−1^ almost all fibres, besides some very thin ones, are (partially) filled with the liquid crystal while the diameter of the deposited fibres grows substantially (Figure 3d). At points where many fibres meet they fuse. This tendency is further increased in the last sample in Figure 3e. The smeared-out morphology in this last texture strongly suggests that the fibre still contained substantial amounts of solvent upon collection. The liquid state of such a deposited material then allowed a post-collection morphology change, ruining the desired coaxial fibre structure.

**Figure 4:**
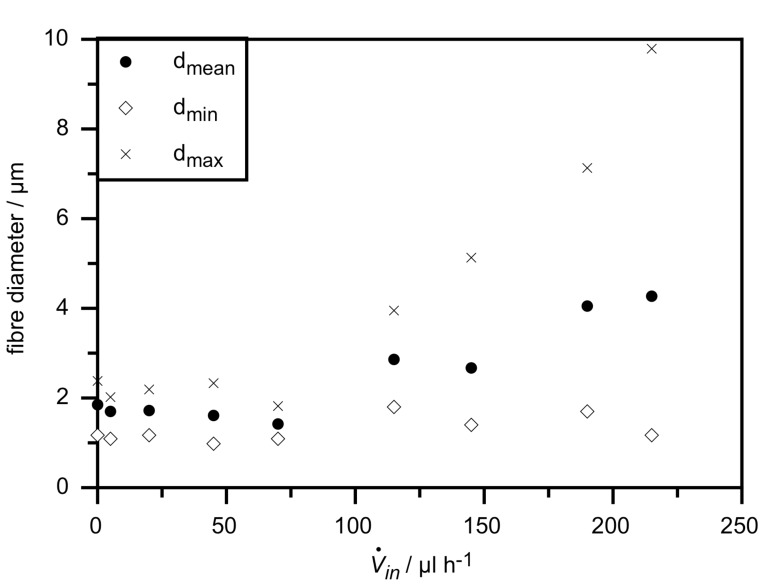
Outer fibre diameters as a function of LC flow rate as determined from 50 measurements by optical microscopy on each sample.

Measuring the exact outer and inner fibre diameter by optical microscopy is quite difficult for such small structures, but we can give reasonably accurate estimates at least for the outer diameter. We determined the mean, minimal and maximal outer diameter of the filled fibres by measuring the diameter at 50 points of each sample. The resulting values are plotted in Figure 4, taking unfilled pure PVP fibres as reference. As one can see, up to the sample with 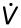
*_in_* = 70 µl h^−1^ all three determined kinds of diameter are unchanged compared to the sample without liquid crystal, within the accuracy of this kind of measurement. With further increasing content of LC core, the smallest found diameters are still in the same range as before, but the mean diameter and especially the largest found diameter increase strongly. In the last samples this may be partly because of the smearing out of the fibres as described at the end of the previous paragraph. But at least for the two highest flow rates the LC core visible in the non-POM photographs is as thick as the whole fibres in the first samples (see for Figure 3d1 and Figure 3e1).

**Figure 5:**
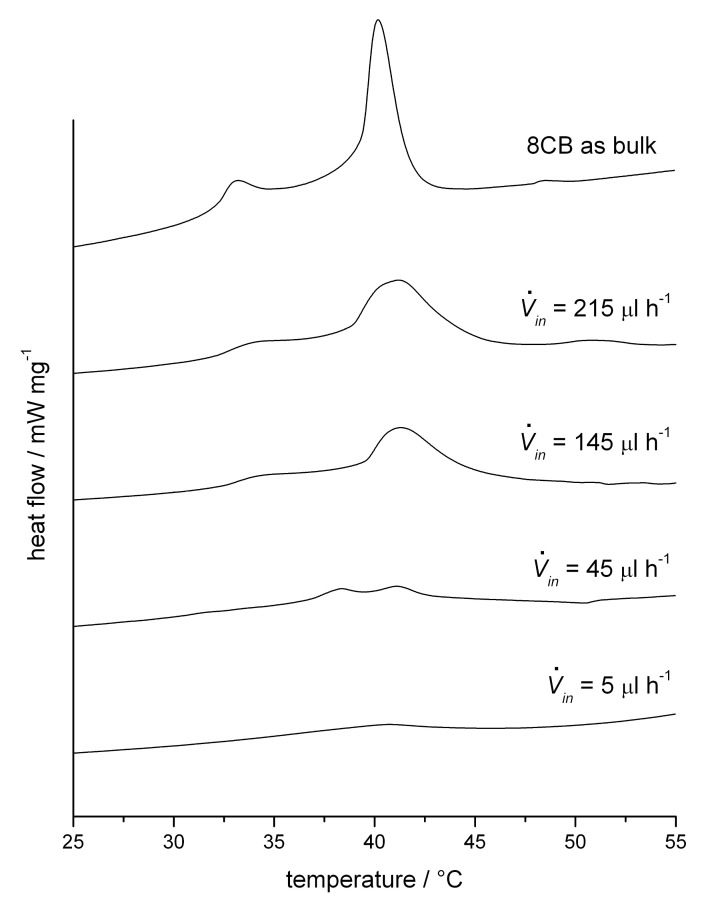
DSC thermograms on heating of 8CB as bulk and as inclusion compound in PVP fibres produced with different flow rates of the LC (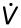
*_in_*).

DSC thermograms of these fibres are presented in Figure 5, as well as the thermogram of bulk 8CB for comparison. The effect of confinement on the LC phase sequence can be clearly seen. In the fibres the clearing peak is broadened and shifted towards higher temperatures compared to the bulk LC, but between the different fibres the clearing point variations are small. The smectic to nematic transition, on the other hand, is shifted to higher temperatures with decreasing LC content of the fibres until it finally disappears in the last shown sample (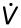
*_in_* = 5 µl h^−1^). During this process the shape of this transition in the thermogram transforms continuously from a peak in the bulk to a step, signifying a change from (weakly) first- to second-order character of the transition.

Another remarkable effect is that in the thermograms obtained for 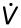
*_in_* = 20 µl h^−1^ and 45 µl h^−1^ during a first heating experiment the SmA-N as well as the clearing transition were visible, but in the cooling run and in a subsequent second heating experiment only the N-iso. transition was found and it was shifted towards lower temperatures. This change in the effective phase sequence could be verified by textural observations during a similar experiment in the microscope. When repeating the DSC heating scan after having kept the sample at room temperature for several weeks, again two peaks were found, just like at the very beginning. We interpret these findings as a strong supercooling effect on the smectic phase, meaning that transformation from the nematic to smectic phase is kinetically hindered in the encapsulated liquid crystal, most likely due to mesogen anchoring without positional order at the inner surface of the polymer sheath. So, after heating into the isotropic phase, on subsequent cooling the smectic phase cannot be formed immediately even though it is the thermodynamically stable phase at room temperature.

Conversely, the same phenomenon explains the increase of the SmA-N transition on initial heating as the fibre diameter is made thinner (Figure 5): if the smectic phase is present in the fibre the layer geometry will be retained even when the temperature is raised somewhat above that where a bulk sample becomes nematic, because the mesogens at the inner sheath surface are anchored in the smectic configuration, with 1D positional order, and the extreme surface-to-volume ratio in a thin fibre gives these surface molecules a much stronger than usual influence on the whole sample behaviour. This reasoning presumes a planar anchoring of the mesogens at the sheath surface, a geometry that was confirmed by X-ray scattering experiments, as described at the end of this article. This finding also correlates with the previously described result that the outer fibre diameter is not changed for a small content of LC core, meaning that only a small fraction of the LC molecules are not affected directly by the polymer surface, so that a strong effect on the LC phase sequence could be expected in this case.

### Influence of applied voltage

According to the numerical study on flow patterns in coaxial electrospinning [15] annular flows with smooth core-sheath interfaces appear only if the electrical field strength is above a certain threshold level. It is also known from another study [12] that the reduction of outer fibre diameter that is generally seen upon increasing field strength is not coupled to any substantial decrease in wall thickness, i.e. the reduction of total fibre diameter is almost completely given by a reduction of core thickness.

**Figure 6:**
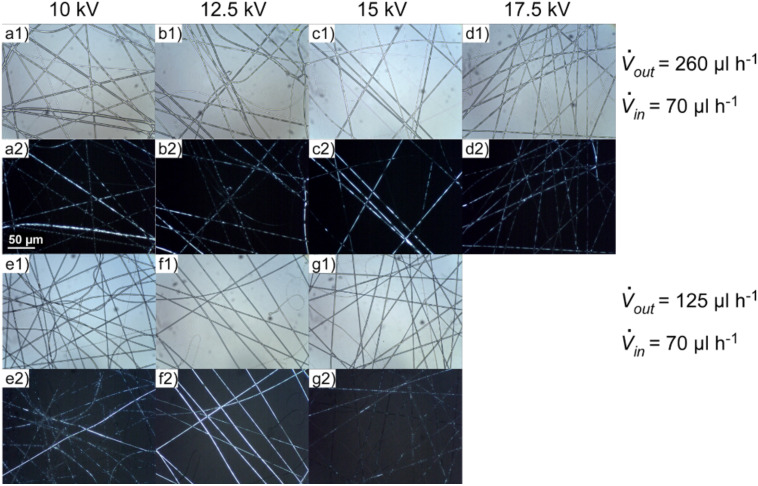
The liquid crystal core shows a transformation from bubbly to smooth and to bubbly again with increasing electric field strength, the threshold voltage being dependent on the ratio of outer to inner flow rate.

Examples of our fibres produced at different field strengths are shown in Figure 6. As one can see, at a combination of outer and inner flow rates of 125 and 70 µl h^−1^, respectively, a smooth inner core is achieved at a voltage of 12.5 kV, while an increase of the outer flow rate to 260 µl h^−1^ (without changing the inner flow rate) requires a voltage of 15 kV. At even higher voltages the filling gets non-uniform again and it becomes more and more difficult to achieve a homogenous fibre mat.

**Figure 7:**
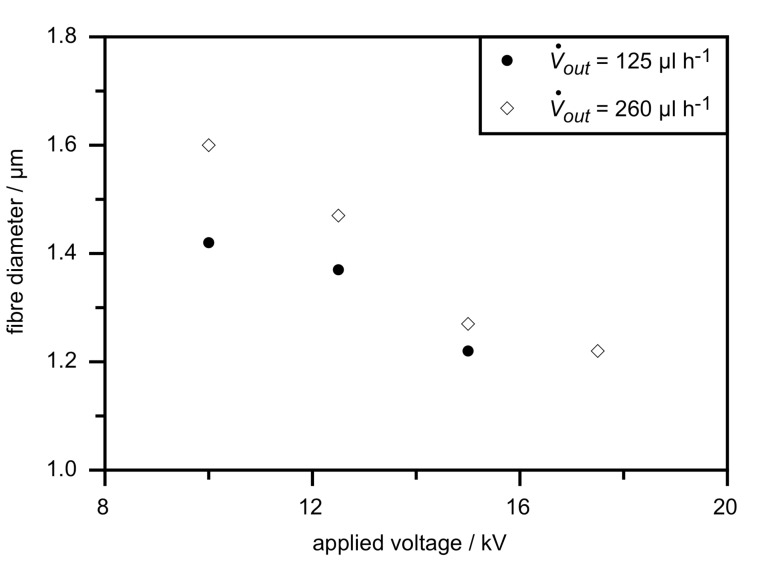
Influence of the applied voltage and the flow rate of the polymer solution on the mean fibre diameter.

In Figure 7 a plot of the dependence on applied voltage of mean fibre diameters measured by optical microscopy of these samples is shown. It is clear from the diagram that the diameters are reduced with increasing field strength and increase with higher flow rate of the polymer outer solution, as is to be expected. But when comparing this reduction of outer fibre diameters to the diameter range found in the previous described experiment with different flow rates of LC (see Figure 4), the variation is rather small. This observation suggests that the effect of the electric field strength variations on the inner diameter is in fact almost negligible, which would explain that we found no influence on the phase transition temperatures, which are the same for all samples shown and whose values fit to the results shown in Figure 5 with corresponding LC flow rate.

Since our observations of the inner diameter are only indirect and would have to be corroborated with direct measurement (practically very difficult to achieve with these particular fibres) our experiments can not be yet fully compared to the results of the above mentioned reference [12] on composites of TiO_2_/PVP sheath with mineral oil as core. We hope to be able to modify our procedures and/or material combinations such that in the future we will be able to investigate the fibres by electron microscopy with respect to the diameter of the LC core.

### X-Ray investigations on fibres

**Figure 8:**
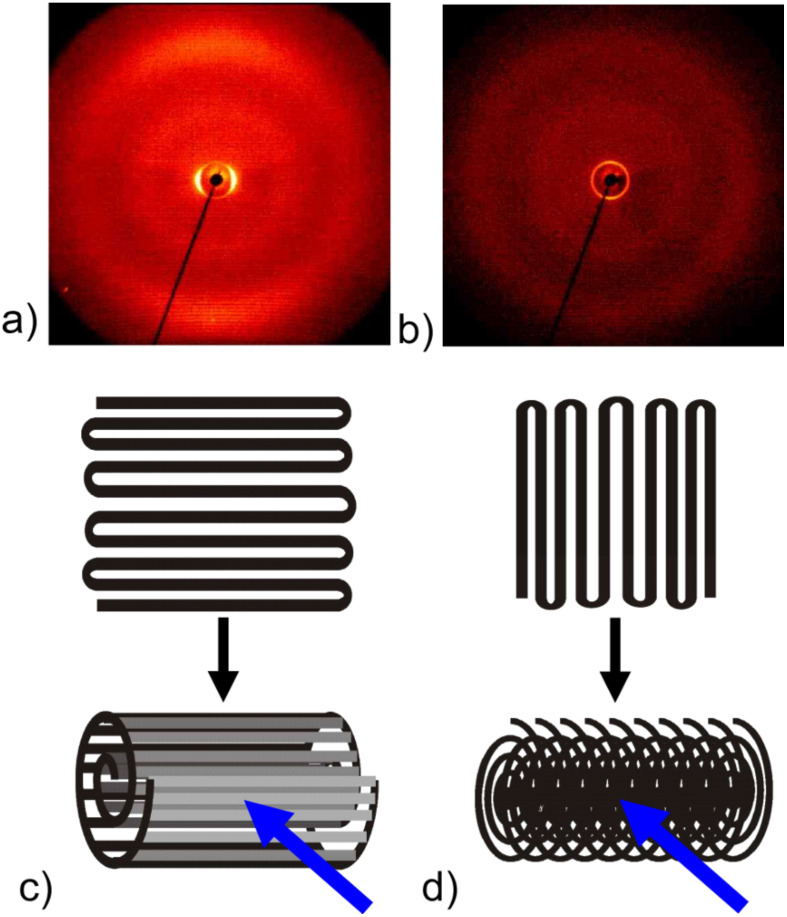
a), b): 2D X-ray diffraction pattern of oriented fibres at two perpendicular roll-up directions schematically sketched in c) and d), respectively. The incident X-ray beam is represented by the blue arrow. These scattering patterns from the composite fibres should be compared to those from unfilled PVP fibres and bulk 8CB, respectively, shown in Figure 9.

To gain information about the orientation of the liquid crystal molecules (and the layers of the SmA phase) inside the fibres, 2D X-ray diffraction patterns were recorded. For this purpose two samples of uniformly aligned fibres with maximum LC content were spun between bars of aluminium foil and were then rolled up in two perpendicular directions. A schematic sketch of the fibre directions before and after rolling is given in Figure 8c and Figure 8d, respectively. The corresponding diffraction patterns can be seen in Figure 8a and Figure 8b, the orientation being more clearly distinguishable in the first pattern than in the second one. This is mainly due to the facts that the roll-up direction can be better controlled in this case and that in the second case the X-ray beam is directed along rather than across the fibres in a large part of the sample, thereby not producing any significant scattering response. So in the following we will discuss Figure 8c in more detail.

**Figure 9:**
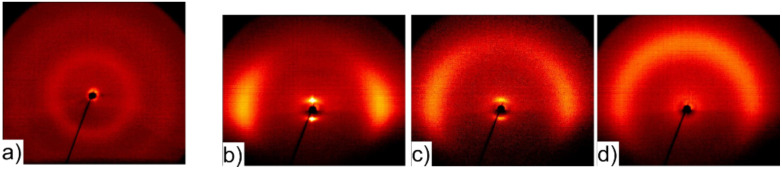
X-ray diffraction patterns of PVP fibres without LC (a) and of surface-aligned samples of bulk 8CB in the SmA, nematic and isotropic phase, respectively (b to d).

The layer reflections of the SmA phase can be seen on the equator in the small angle region. The corresponding layer spacing d_A_ = 3.08 nm can be very well compared to the value of 3.14 nm measured for bulk 8CB (Figure 9b) and to literature values (see for example [16–17]). In the middle and wide angle regions of the diffractogram two diffuse rings can be seen, caused by the polymer sheath (compare to Figure 9a). The outer diffuse ring is on the meridian overlayed by diffuse scattering due to the liquid-like distributed lateral distance between the LC molecules.

**Figure 10:**
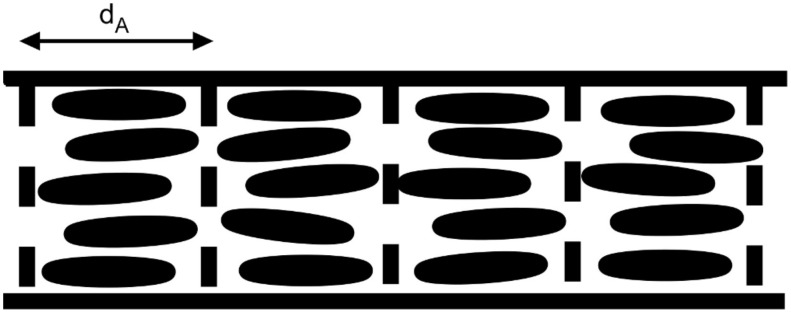
Schematic sketch of the arrangement of LC molecules and the smectic layers in an electrospun fibre.

When considering the orientation of the fibres with respect to the incident X-ray beam the finding of layer reflections on the equator and of transverse spacing peaks on the meridian leads to a model in which the smectic layers are arranged perpendicular to the fibre formation axis (cf. Figure 10). This is somewhat unexpected, since the fibres are spun at a temperature at which the liquid crystal is in its smectic phase, in which the lowest viscosity is along the layers and not perpendicular to them. Considering only the flow geometry [18–19] one would thus expect concentric layers extended along the fibre axis in contrast to our experimental results. Possible explanations of this discrepancy might be either that the significant stretching during the electrospinning process leads to a shear induced phase transition to the nematic phase, causing a rearrangement of the molecules, or that the molecules are rearranged after electrospinning as a result of the interaction with the inner PVP sheath surface.

## Conclusion

We show in this work that a liquid crystal in its smectic phase can be embedded as core in a PVP microfibre via coaxial electrospinning resulting in a new composite material with interesting properties. In our system the behaviour of the LC could be investigated by calorimetry measurements, X-ray diffraction experiments and by polarized optical microscopy, the latter one being possible only because the polymer we used as sheath material is transparent and isotropic, which is an improvement to previous work with PVP/TiO_2_ as sheath [13]. From the X-ray diffraction patterns determined on oriented fibres we can conclude that in our fibres at room temperature the smectic phase still exists with the smectic layers being oriented perpendicular to the fibre formation axis, an unexpected result when considering the flow geometry of a smectic phase.

By varying the relation between the flow rate of the outer polymer solution and the flow rate of the LC the morphology of the core could be changed stepwise from discontinuous to very thin but continuous with overall fibre thicknesses comparable to unfilled fibres. In this regime complex phase behaviour dictated by strong surface effects was found. At higher LC flow rates a continuous bulk-like core and thick fibres were found. On the other hand, by increasing the voltage applied during electrospinning over a certain threshold value (dependent on the outer flow rate) a discontinuous core could be transformed into a continuous one while reducing the fibre diameter only slightly so that the phase behaviour was not influenced by this parameter. So by combining these two variables, maybe also with others relevant for electrospinning like the concentration of the polymer solution, composite fibres with the desired properties can be produced.

## Experimental

The fibres were spun from a solution of 12.5 wt % PVP (Acros, MW 1,300,000 g/mol) and 0.5 wt % NaCl in ethanol through an outer tube of 0.7 mm inner diameter. The core liquid crystal 8CB (Synthon Chemicals GmbH) was pumped at room temperature through an inner capillary of 320 μm inner and 450 μm outer diameter (fused silica tubing, BGB Analytik AG). The spinneret–collector distance was kept constant at 10 cm. With a high voltage power supply (Gamma High Voltage Research) a voltage of 10 kV was applied, unless otherwise stated. All fluids were pumped at a controlled rate using a Fluigent MFCS4C microfluidics controller. The resulting samples were kept at room temperature for several days prior to conducting DSC or X-ray studies to ensure that all solvent was evaporated so that the fibres were mechanically stable. Oriented fibres for X-ray investigations were spun across parallel bars of aluminium foil placed on the collector.

Calorimetry investigations were carried out on a Perkin Elmer Pyris 1 DSC at a heating rate of 10 K min^−1^. Samples of about 5 to 15 mg fibres were used for the measurements. X-ray investigations of fibres were performed on oriented fibres, rolled up to achieve a compact sample (see Figure 8c and Figure 8d). The sample of the bulk liquid crystal was aligned at the sample–air interface while cooling slowly on a glass plate, the temperature-controlled heating stage partially shadowing the patterns below the equator. The diffraction patterns were recorded with a 2D detector (HI-STAR, Siemens) using Ni-filtered CuK_α_ radiation.
